# The Combination of Functional and Structural MRI Is a Potential Screening Tool in Alzheimer’s Disease

**DOI:** 10.3389/fnagi.2018.00251

**Published:** 2018-09-21

**Authors:** Chun-Chao Huang, Wei-Ming Huang, Chia-Hung Chen, Zong-Yi Jhou, The Alzheimer’s Disease Neuroimaging Initiative, Ching-Po Lin

**Affiliations:** ^1^Institute of Neuroscience, National Yang-Ming University, Taipei, Taiwan; ^2^Department of Radiology, Mackay Memorial Hospital, Taipei, Taiwan; ^3^Department of Medicine, Mackay Medical College, Taipei, Taiwan; ^4^Mackay Junior College of Medicine, Nursing, and Management, Taipei, Taiwan; ^5^Department of Biomedical Imaging and Radiological Sciences, National Yang-Ming University, Taipei, Taiwan; ^6^Brain Research Center, National Yang-Ming University, Taipei, Taiwan

**Keywords:** mild cognitive impairment, Alzheimer’s disease, CSF biomarkers, FDG-PET, resting-state functional MRI, structural MRI

## Abstract

**Introduction:** This study aimed to survey the discrimination power of parameters from cerebrospinal fluid (CSF) biomarkers, fluorodeoxyglucose uptake on PET (FDG-PET), structural magnetic resonance imaging (MRI), and functional MRI in high- and low-risk subjects or in converters and stable subjects of normal and mild cognitive impairment (MCI) statuses.

**Methods:** We used baseline resting-state functional MRI (rfMRI) from the Alzheimer’s Disease Neuroimaging Initiative (ADNI) dataset to analyze functional networks and recorded subjects’ characteristics and results of the CSF study, FDG-PET, and structural MRI from the ADNI website. All parameters were evaluated based on the between-group difference among normal (NC), MCI, and Alzheimer’s disease (AD) groups. The parameters other than CSF results were included to study the difference between high- and low-AD-risk subjects in NC or MCI groups, based on CSF results. On the basis of two-year follow-up conditions, all parameters were compared between stable subjects and converters in NC and MCI.

**Results:** CSF biomarkers, FDG-PET, structural MRI, and functional MRI are all able to differentiate AD from MCI or NC but not between MCI and NC. As compared with low-AD-risk subjects, high-risk subjects present decreased FDG-PET in both MCI and NC groups but structural MRI change only in MCI status and rfMRI alteration only in NC status. As compared with stable subjects, converters have decreased FDG-PET, functional network changes, and structural changes in both MCI and NC groups.

**Conclusion:** The combination of functional and structural MRI is a safer screening tool but with similar power as FDG-PET to reflect CSF change in the AD pathological process and to identify high-risk subjects and converters in NC and MCI.

## Introduction

Alzheimer’s disease (AD) is the most common form of dementia, affecting more than 10% elder people, and is one of the most prevalent problems with significant economic burdens in the aging society all over the world, which is predicted to be even worse in the near future (Sperling et al., 2011b; Bateman et al., 2012). Treatment for AD has been widely explored, but disappointingly, effective disease-modifying treatments are currently not yet available (Sperling et al., 2011b; Dubois et al., 2014). The underlying neuropathological changes of AD are intraneuronal neurofibrillary tangles and extracellular neuritic plaques. The main component of these plaques is amyloid-beta (Aβ) peptide, and antiamyloid treatments are the mainstream in the field of management of AD but fail to improve clinical outcomes (Doody et al., 2014; Salloway et al., 2014). Considering that the pathological process begins to occur years or even decades before clinical diagnosis of AD (Hulette et al., 1998; Price and Morris, 1999; Price et al., 2009), the goal of therapeutic strategies is currently focused on prevention with intervention initiated as early as possible, even in preclinical stage (Cummings et al., 2007; Fagan et al., 2011; Sperling et al., 2011b). The achievement of this goal relies on specific biomarkers, which can early detect subjects at risk. In this field, many potential biomarkers have been proposed predominantly from results of cerebrospinal fluid (CSF), plasma, and structural and functional neuroimaging (Shaw et al., 2007; Hampel et al., 2008). Among all, five biomarkers are currently considered more powerful, including decreased CSF Aβ_42_, increased CSF tau, decreased fluorodeoxyglucose uptake on PET (FDG-PET), PET amyloid imaging, and brain atrophy on structural magnetic resonance imaging (MRI) (Jack et al., 2010; Dubois et al., 2014). With increasing supporting evidence, PET amyloid imaging and the combination change of CSF Aβ_42_ and tau are included in the criteria to diagnose AD or to detect subjects at risk (Dubois et al., 2014).

Screening for subjects with risk for AD is commonly performed by means of memory tests. However, subjects identified by these tests might already be in the status of mild cognitive impairment (MCI) or only 2 to 4 years before onset of AD (Derby et al., 2013). Although memory impairment is one of the earliest signs for AD, this symptom occurs much later than above-mentioned biomarkers. During the pathological process of AD, the first two biomarkers with detectable change are CSF Aβ_42_ and PET amyloid, then CSF tau and FDG-PET, and finally structural MRI (Jack et al., 2010). However, the CSF study is invasive and the PET study is expensive and with radiation hazard. Even with many reports of MR-related injuries, MRI is still considered very safe as long as safety guidelines are strictly followed to perform this procedure (Shellock and Crues, 2004). Although MRI is not inexpensive, considering safety and early identification, MRI might be the better screening method than the other biomarkers and memory tests, but the power of early detection should be enhanced in order the maximize the screening value because the intervention of AD should be initiated as early as possible. For this reason, we try to evaluate the values of structural MRI and another relatively less emphasized biomarker, functional MRI, in the prediction of the AD pathological process as compared with other more validated earlier biomarkers, including FDG-PET and CSF studies. In addition, risk stratification is applied to see whether AD-related changes are different between high- and low-risk subjects, and further follow-up results are included to detect possible indicators with more reliable power to differentiate converters from stable subjects.

## Materials and Methods

### Participants

Data used in the preparation of this article were obtained from the Alzheimer’s Disease Neuroimaging Initiative (ADNI) database (adni.loni.usc.edu). The ADNI was launched in 2003 as a public–private partnership, led by Principal Investigator Michael W. Weiner, MD. The primary goal of ADNI has been to test whether serial MRI, PET, other biological markers, and clinical and neuropsychological assessment can be combined to measure the progression of MCI and early AD. Subjects with baseline resting-state functional MRI (rfMRI) data and preprocessed volumetric three-dimensional magnetization prepared rapid acquisition gradient echo (3D-MPRAGE) T1-weighted images were included and then left-handed subjects were excluded. According to the baseline diagnosis, all subjects were separated into three diagnostic groups: normal (NC), MCI, and AD. Based on the selection criteria from ADNI, the MCI subjects had reported a subjective memory concern autonomously or via an informant or clinician; however, daily living activities were preserved and neither significant impairment in other cognitive domains nor signs of dementia existed. From the ADNI dataset, baseline data of the following parameters for each subject were recorded: age, gender, years of schooling, Mini-Mental State Examination (MMSE), CSF Aβ_42_ concentration, CSF total tau (T-tau) concentration and CSF phosphorylated tau (P-tau) concentration, FDG-PET results, hippocampal volume, whole-brain volume (WholeBrain), and total intracranial volume (ICV). The FDG-PET results were individual whole-brain average uptake values, standardized uptake values (SUVs), from the published ADNI dataset, analyzed by one of core labs of ADNI. Furthermore, we calculated the ratio of T-tau to Aβ_42_ (T-tau/Aβ_42_), P-tau to Aβ_42_ (P-tau/Aβ_42_), hippocampal volume to whole-brain volume (Hippo/WholeBrain), and hippocampal volume to ICV (Hippo/ICV). Brain atrophy was measured by the following: (1 – WholeBrain/ICV) × 100%.

### MRI Acquisition and Analysis

All raw baseline rfMRI data and preprocessed 3D-MPRAGE T1-weighted images were downloaded from the public ADNI website. The rfMRI data were preprocessed using FSL v5.0.8 (Functional Magnetic Resonance Imaging of the Brain Software Library^1^) (Smith et al., 2004; Woolrich et al., 2009; Jenkinson et al., 2012). The preprocessing steps applied to these images were the following: slice timing correction, motion correction, removing non-brain tissue, smoothing, and high-pass temporal filtering to remove low-frequency drifts. Then, the preprocessed time series data were registered into a stereotactic space (MNI152 template; Montreal Neurological Institute [MNI], Montreal, QC, Canada) (Jenkinson and Smith, 2001; Greve and Fischl, 2009), and the MNI-space time series data were resampled to 4-mm resolution for group independent component analysis (ICA) analysis to generate intrinsic functional network templates with dimensionality at 32, which was from automatic dimensionality estimation.

Using fslcc utility in FSL to compare with resting-state network (RSN) templates (Smith et al., 2009), 10 RSNs were then identified visually from the ICA components: (1) visual medial, (2) visual occipital, (3) visual lateral, (4) default mode network (DMN), (5) cerebellum, (6) sensorimotor, (7) auditory, (8) executive control, (9) right frontoparietal, and (10) left frontoparietal networks. Individual functional networks were calculated by using the dual regression approach. Then, functional strength (FS) for each functional network was calculated by averaging connectivity strength across all voxels within each RSN template mask for each subject. In addition, functional connectivity (FC) for DMN was evaluated by calculating the correlation among mean time courses of the resultant four core clusters of DMN.

### Statistical Analysis

Statistical analysis was performed using the Statistical Package for Social Sciences (SPSS) program, version 20 (SPSS Inc., Chicago, IL, United States). After test of homogeneity of variance for all parameters among the three diagnostic groups, NC, MCI, and AD, the one-way analysis of variance with the *post hoc* test using Scheffe’s method to observe group differences was applied for the parameters with equal population variances. For parameters without equal population variances, the Brown–Forsythe test was conducted with the *post hoc* test using the Dunnett T3 test. In addition, subjects in NC and MCI groups were further divided into two subgroups with above or below within group average of T-tau/Aβ_42_ or P-tau/Aβ_42_, respectively. Then, the independent two-sample *t* test was performed for the rest of the parameters. The separation based on the average ratio of tau protein and Aβ_42_ was not reliable because the average ratio would vary a lot when using another subject group. However, there were still no specific values of these ratios to define high-risk subjects in the literature. We used averages as the cut point only for the ease of statistical analysis, but this method had limitations definitely. Finally, based on two-year follow-up clinical conditions, subjects with converting status from NC to MCI or from MCI to AD were identified and parameters of these subjects were compared with those of stable subjects in the corresponding group by means of the independent two-sample *t* test. Fisher’s exact test was also performed for the relation between high- or low-risk subjects and stable subjects or converters in NC and MCI groups. The threshold for statistical significance was a *p*-value of less than 0.05.

## Results

### Subjects

With the initial inclusion criteria, 173 subjects were selected from the ADNI database. Among them, 43 subjects were excluded for the following reasons: 11 left-handed, 5 failing to transform fMRI data to a 4D image, 2 with marked motion artifact, 10 with imaging distortion, and 15 without CSF data. The remaining 130 subjects were divided into three groups based on the baseline diagnosis: NC group: 41 subjects with 17 men, MCI group: 64 subjects with 34 men, and AD group: 25 subjects with 13 men. These three groups did not show any statistically difference in age, gender, and years of schooling (**Table 1**).

**Table 1:** The characteristics and data among the three diagnostic groups: NC, MCI, and AD.

Group (subject number)	NC (41)	MCI (64)	AD (25)	*p*-value
**Demographic and others**		
Age (years)	73.88 ± 5.89	71.05 ± 7.41	72.94 ± 7.43	
Gender (M:F)	17:24	34:30	13:12	
Education (years)	16.71 ± 1.99	15.92 ± 2.60	15.52 ± 2.63	
MMSE	28.78 ± 2.78 (a)	28.00 ± 1.75 (b)	22.96 ± 2.35 (a,b)	**a: < 0.001, b: < 0.001**
FDG-PET	6.66 ± 0.50 (a)	6.41 ± 0.54 (b)	5.25 ± 0.65 (a,b)	**a: < 0.001, b: < 0.001**
**CSF results**		
Aβ42 (pg/mL)	190.74 ± 55.26 (a)	185.08 ± 56.87 (b)	140.58 ± 40.18 (a,b)	**a: < 0.001, b: < 0.001**
T-tau (pg/mL)	72.36 ± 33.74 (a)	84.46 ± 52.53 (b)	133.47 ± 79.02 (a.b)	a:0.003, b:0.021
P-tau (pg/mL)	38.47 ± 25.85 (a)	42.15 ± 23.15 (b)	56.93 ± 26.82 (a,b)	a:0.015, b:0.044
T-tau/Aβ42	0.44 ± 0.32 (a)	0.56 ± 0.54 (b)	1.06 ± 0.80 (a,b)	a:0.003, b:0.021
P-tau/Aβ42	0.24 ± 0.24 (a)	0.27 ± 0.20 (b)	0.45 ± 0.30 (a,b)	a:0.003, b:0.006
**Structural MRI**		
Hippocampus (cm3)	7.80 ± 0.95 (a)	7.43 ± 1.01 (b)	6.05 ± 1.05 (a,b)	**a: < 0.001, b: < 0.001**
Entorhinal (cm3)	3.96 ± 0.68 (a)	3.73 ± 0.73 (b)	2.95 ± 0.72 (a,b)	**a: < 0.001, b: < 0.001**
Fusiform (cm3)	18.22 ± 2.13 (a)	18.03 ± 2.25 (b)	16.08 ± 2.41 (a,b)	a:0.002, b:0.002
MidTemp (cm3)	20.51 ± 3.01 (a)	20.43 ± 2.42 (b)	17.71 ± 2.72 (a,b)	**a:0.001, b: < 0.001**
WholeBrain (cm3)	1080 ± 121	1061 ± 100	1026 ± 128	
ICV (cm3)	1567 ± 185	1551 ± 157	1581 ± 214	
Hippo/WholeBrain (%)	0.73 ± 0.06 (a)	0.70 ± 0.08 (b)	0.61 ± 0.08 (a,b)	**a: < 0.001, b: < 0.001**
Hippo/ICV (%)	0.50 ± 0.06 (a)	0.48 ± 0.07 (b)	0.39 ± 0.06 (a,b)	**a: < 0.001, b: < 0.001**
Ento/WholeBrain (%)	0.37 ± 0.05 (a)	0.35 ± 0.06 (b)	0.29 ± 0.07 (a,b)	**a:0.001, b: < 0.001**
Ento/ICV (%)	0.26 ± 0.04 (a)	0.24 ± 0.05 (b)	0.19 ± 0.04 (a,b)	**a:0.001, b: < 0.001**
Fusi/WholeBrain (%)	1.70 ± 0.16 (a)	1.69 ± 0.15 (b)	1.59 ± 0.18 (a,b)	a:0.031, b:0.028
Fusi/ICV (%)	1.18 ± 0.12 (a)	1.16 ± 0.12 (b)	1.02 ± 0.13 (a,b)	**a:0.001, b: < 0.001**
MidT/WholeBrain (%)	1.90 ± 0.17 (a)	1.92 ± 0.15 (b)	1.75 ± 0.19 (a,b)	a:0.002, **b: < 0.001**
MidT/ICV (%)	1.32 ± 0.13 (a)	1.32 ± 0.13 (b)	1.13 ± 0.15 (a,b)	**a: < 0.001, b: < 0.001**
Brain atrophy (%)	30.79 ± 3.78 (a)	31.47 ± 3.43 (b)	35.57 ± 3.00 (a,b)	**a: < 0.001, b: < 0.001**
**Functional MRI**		
FC of DMN	0.62 ± 0.13	0.59 ± 0.13	0.54 ± 0.17	
Visual medial	5.79 ± 1.47	6.01 ± 1.23	5.59 ± 1.14	
Visual occipital	1.35 ± 0.46	1.29 ± 0.40	1.34 ± 0.45	
Visual lateral	2.17 ± 1.05	2.36 ± 1.18	2.11 ± 1.34	
DMN	6.17 ± 1.27	6.48 ± 1.34 (a)	5.63 ± 1.64 (a)	a:0.036
Cerebellum	2.00 ± 0.66	1.91 ± 0.63	1.96 ± 0.54	
Sensorimotor	2.93 ± 1.16	2.92 ± 1.27	2.79 ± 1.22	
Auditory	3.21 ± 0.84 (a)	3.31 ± 0.89 (b)	2.77 ± 0.55 (a,b)	a:0.039, b:0.003
Executive control	2.45 ± 1.13	2.44 ± 0.90	2.73 ± 1.24	
Right frontoparietal	2.99 ± 0.92	3.17 ± 0.75	3.00 ± 0.97	
Left frontoparietal	2.62 ± 0.96	2.71 ± 0.68	2.33 ± 0.81	

*The denotation of “a” and “b” means the between-group difference with the corresponding *p*-value. The *p*-values in bold represent the tests that pass multiple-comparison correction with the adjusted *p*-value of 0.0014.*

*Abbreviations: NC, normal; MCI, mild cognitive impairment; AD, Alzheimer’s disease; MMSE, Mini-Mental State Examination; FDG-PET, fluorodeoxyglucose uptake on PET; WholeBrain, whole-brain volume; ICV, intracranial volume; Hippo, hippocampus; FC, functional connectivity; DMN, default mode network.*

### Group Differences Among NC, MCI, and AD

The results of all parameters in these three groups are listed in **Table 1**. For the CSF data, a significantly lower value of Aβ_42_ and higher values of T-tau, P-tau, P-tau/Aβ_42_, and T-tau/Aβ_42_ were noted in AD as compared with NC or MCI; however, there was no significant difference of the above parameters between NC and MCI. The FDG-PET result showed a significantly lower value in AD as compared with NC or MCI, but there was no significant difference between NC and MCI. For the structural MRI results, hippocampus, Hippo/WholeBrain, and Hippo/ICV showed similar results as FDG-PET. There was more brain atrophy in AD than NC or MCI, but there was no difference between NC and MCI. For the functional MRI results, a significant difference was noted only in FS of DMN and auditory network. In DMN, FS in AD was lower than that in MCI, but there was no difference between AD and NC or between MCI and NC. In the auditory network, FS in AD was lower than that in NC or in MCI, but there was no difference between NC and MCI. As shown in **Table 1**, a total of 36 comparisons were evaluated, and therefore, considering the multiple-comparison problem, the adjusted *p*-value should be 0.0014. The tests passing multiple-comparison correction are represented by bold *p*-values. Other tests without passing multiple-comparison correction should be interpreted with caution.

### Within-Group Differences Between High and Low T-tau/Aβ_42_ or P-tau/Aβ_42_ in NC or MCI

Significant results are detailed in **Table 2**. The high T-tau/Aβ_42_ group revealed a significantly lower FDG-PET value in NC and MCI, significantly decreased FS of the visual occipital network, visual lateral network, and DMN in NC, and a significantly lower value of Hippo/WholeBrain, Hippo/ICV, Ento/WholeBrain, MidT/WholeBrain, and MidT/ICV in MCI. The high P-tau/Aβ_42_ group displayed higher FS of the left frontoparietal network in NC and a lower FDG-PET value in MCI.

**Table 2:** Within-group differences between high and low T-tau/Aβ_42_ or P-tau/Aβ_42_ in NC or MCI groups.

	T-tau/Aβ42			P-tau/Aβ42		
Parameter (group)	High (14)	Low (27)	*p*-value	High (11)	Low (30)	*p*-value
FDG-PET (NC)	6.40 ± 0.44	6.79 ± 0.48	0.015			
Visual occipital (NC)	1.10 ± 0.47	1.49 ± 0.41	0.010			
Visual lateral (NC)	1.63 ± 0.78	2.45 ± 1.08	0.017			
DMN (NC)	5.62 ± 1.35	6.45 ± 1.15	0.044			
Left frontoparietal (NC)				3.12 ± 1.04	2.44 ± 0.88	0.044

**Parameter (group)**	**High (23)**	**Low (41)**	***p*-value**	**High (23)**	**Low (41)**	***p*-value**

FDG-PET (MCI)	6.20 ± 0.57	6.53 ± 0.50	0.020	6.20 ± 0.57	6.53 ± 0.50	0.020
Hippo/WholeBrain % (MCI)	0.67 ± 0.08	0.73 ± 0.08	0.006			
Hippo/ICV % (MCI)	0.45 ± 0.07	0.50 ± 0.07	0.011			
Ento/WholeBrain (%) (MCI)	0.32 ± 0.08	0.36 ± 0.05	0.013			
MidT/WholeBrain (%) (MCI)	1.86 ± 0.14	1.95 ± 0.15	0.033			
MidT/ICV (%) (MCI)	1.27 ± 0.13	1.34 ± 0.12	0.042			


*The numbers in parentheses after High or Low are subject number.*

*Abbreviations: NC, normal; MCI, mild cognitive impairment; FDG-PET, fluorodeoxyglucose uptake on PET; DMN, default mode network; Hippo, hippocampus; WholeBrain, whole-brain volume; ICV, intracranial volume.*

### Within-Group Differences Between Stable Subjects and Converters in NC or MCI and the Relation Between Risk and Two-Year Follow-Up Status

Significant results are detailed in **Table 3**. In NC, converters had relatively older age, higher T-tau/Aβ_42_, P-tau/Aβ_42_, lower FDG-PET value, lower Hippo/ICV, more brain atrophy, and increased FS of the sensorimotor network. In MCI, converters had relatively higher T-tau/Aβ_42_, P-tau/Aβ_42_, lower FDG-PET value, and decreased FS of the executive control network.

**Table 3:** Differences between stable subjects and converters in NC and MCI groups within 2 years.

	Within 2 years		
NC	Stable (32)	Converter (4)	*p*-value
Age	73.64 ± 5.73	79.88 ± 5.28	0.047
T-tau/Aβ42	0.36 ± 0.24	0.76 ± 0.43	0.007
P-tau/Aβ42	0.18 ± 0.13	0.41 ± 0.24	0.004
FDG-PET	6.76 ± 0.41	5.96 ± 0.30	0.001
Hippo/ICV (%)	0.51 ± 0.06	0.43 ± 0.03	0.01
Brain atrophy (%)	30.22 ± 3.51	36.12 ± 2.25	0.003
Sensorimotor	2.90 ± 1.15	4.12 ± 0.89	0.048

**MCI**	**Stable (37)**	**Converter (9)**	***p*-value**

T-tau (pg/mL)	84.47 ± 51.19	127.83 ± 74.01	0.043
P-tau (pg/mL)	42.58 ± 23.45	64.80 ± 20.89	0.013
T-tau/Aβ42	0.56 ± 0.50	1.00 ± 0.86	0.045
P-tau/Aβ42	0.28 ± 0.20	0.47 ± 0.24	0.018
FDG-PET	6.49 ± 0.47	5.83 ± 0.56	0.001
Executive control	2.62 ± 0.88	1.88 ± 0.61	0.022
MidTemp (cm3)	20.69 ± 2.46	18.72 ± 1.96	0.033


*Abbreviations: NC, normal; MCI, mild cognitive impairment; FDG-PET, fluorodeoxyglucose uptake on PET; Hippo, hippocampus; ICV, intracranial volume.*

In NC, there were eight subjects with both higher T-tau/Aβ_42_ and P-tau/Aβ_42_, and three of them converted to the MCI condition within two-year follow-up. In contrast, only 1 of the remaining 28 subjects converted to the MCI condition. Fisher’s exact test revealed a significant difference with a *p*-value of 0.028. In MCI, there were 18 subjects with both higher T-tau/Aβ_42_ and P-tau/Aβ_42_, and 7 of them converted to the AD condition within 2-year follow-up. In contrast, only 2 of the rest 28 subjects converted to the AD condition. Fisher’s exact test revealed a significant difference with a *p*-value of 0.018.

## Discussion

Among the three diagnostic groups, many parameters can be used to differentiate AD from NC or MCI, including CSF data, FDG-PET, hippocampal volume and brain atrophy in structural MRI, and FS of DMN and auditory network in functional MRI. However, there is no significantly different parameter between NC and MCI. After divided into subjects with high or low risk in NC or MCI by referring to T-tau/Aβ_42_ or P-tau/Aβ_42_, high-risk subjects in NC are with a lower value of FDG-PET and significant differences of rfMRI results, whereas high-risk subjects in MCI are with a lower value of FDG-PET and significant differences of structural MRI results. Subjects in NC or MCI with higher T-tau/Aβ_42_ and P-tau/Aβ_42_ tend to convert to MCI or AD, respectively. The converters in both NC and MCI are with higher T-tau/Aβ_42_, P-tau/Aβ_42_, lower FDG-PET value, and rfMRI change but older age and structural MRI changes are only found in converters in NC.

The frustration of current AD management with antiamyloid treatments is thought to be due to late intervention while the pathological process of AD starts a couple of years before onset of symptoms (Hulette et al., 1998; Price and Morris, 1999; Cummings et al., 2007; Price et al., 2009; Fagan et al., 2011; Sperling et al., 2011b; Doody et al., 2014; Salloway et al., 2014). MCI is a clinical transitional status between the cognitively normal condition and AD, but the diagnosis of MCI is still in the late stage of the overall pathological process (Jack et al., 2010). Therefore, many biomarkers have been explored to survey the power in the early identification of the preclinical insidious process of AD (Shaw et al., 2007; Hampel et al., 2008). Among all, five more reliable biomarkers are commonly used and their changes occur chronologically. Initially, PET amyloid imaging and decreased CSF Aβ_42_ are able to detect Aβ-plaque deposition. Then, increased CSF tau and decreased FDG-PET can reveal the condition of neuronal injury and brain dysfunction. Finally, brain atrophy on structural MRI occurs just before impairment of memory (Jack et al., 2010; Dubois et al., 2014). Previous studies suggested that before brain atrophy, Aβ-plaque deposition accumulates to a significantly high level far before the onset of AD and the change might only be slight without a significant difference among normal subjects with high Aβ-plaque deposition, MCI, and AD (Engler et al., 2006; Jack et al., 2009). CSF tau and FDG-PET display the pathological process of AD after Aβ-plaque deposition and the changes are more obvious between the normal condition and MCI but become less distinct between MCI and AD (Minoshima et al., 1997; Sunderland et al., 1999; Bouwman et al., 2007; Vemuri et al., 2009a). Functional MRI is able to reflect synaptic dysfunction and is thought to display a similar pathological process as FDG-PET in AD (Sperling et al., 2011a). Our data do not have PET amyloid imaging results, but extremely high concordance exists between low CSF Aβ_42_ and positive PET amyloid imaging, and T-tau/Aβ_42_ and P-tau/Aβ_42_ show excellent predictive power for high amyloid-plaque deposition (Fagan et al., 2006; Tolboom et al., 2009; Fagan et al., 2011). However, in our study, all the changes of CSF Aβ_42_, tau, FDG-PET, and functional MRI demonstrate a significant difference only between AD and NC or MCI but not between NC and MCI. Structural MRI is with greater power to reflect the late pathological process in AD than above biomarkers and is predictive of future conversion from MCI to AD, especially the hippocampal volume (Fox and Freeborough, 1997; Vemuri et al., 2009b; Bateman et al., 2012). Our results are compatible with this phenomenon, showing decreased hippocampal volume and more brain atrophy in AD than NC or MCI but no difference between NC and MCI. The change of CSF data, FDG-PET, and rfMRI are considered more obvious between NC and MCI than between MCI and AD. However, significant differences of the above parameters are only found between AD and NC or MCI in our study. The differences of the above parameters between AD and the other two groups are still reasonable because although changes of these parameters start early from NC, they persist across the status of MCI to AD (Jack et al., 2010; Sperling et al., 2011a). The surprising point is that they do not differ from each other between NC and MCI. Considering the characteristics of the included subjects, one possible explanation is that the subjects in NC were old enough to be at high risk for AD or already in the AD pathological process. The viewpoint is supported by the two-year follow-up results, in which the converters from NC to MCI are not rare within just 2 years.

We further divide subjects in NC and MCI into low and high AD risk to evaluate the significant biomarker change in these two statuses. Currently, asymptomatic people with two biomarkers are considered at risk for AD. One biomarker is decreased Aβ_42_ together with increased T-tau or P-tau in CSF and the other one is positive PET amyloid imaging (Dubois et al., 2014). These two biomarkers are highly correlated with each other and T-tau/Aβ_42_ and P-tau/Aβ_42_ are excellently predictive of amyloid-positive people (Fagan et al., 2011). Because of lack of amyloid PET results, we use the averages of these two ratios to divide subjects in NC and MCI into subjects with relatively high or low risk. From our results, FDG-PET shows decreased metabolism in high-risk subjects in both NC and MCI, suggesting that the process of impaired synaptic function exists in these two statuses and indicating that subjects at risk convert to next pathological status, compatible with the results of previous studies (de Leon et al., 2001; Jack et al., 2010). Structural MRI reveals only differences in MCI group with lower Hippo/WholeBrain, Hippo/ICV, Ento/WholeBrain, MidT/WholeBrain, and MidT/ICV values in high-risk subjects. As compared with whole-brain atrophy and hippocampal volume, the relative volume ratio of hippocampus and whole brain or ICV is more sensitive to reflect high-risk subjects in our study. The hippocampal volume is the more sensitive measurement in the structural MRI for AD pathological change (Bateman et al., 2012). In our study, only Hippo/WholeBrain and Hippo/ICV but not hippocampus *per se* are able to differentiate high- and low-risk subjects in MCI group, probably because these ratios exclude the effect of the between-subject brain volume difference and represent hippocampal shrinkage more precisely. Furthermore, these structural changes occur only in MCI group but not in NC group, consistent with previous literature suggesting that structural change is a late biomarker with more atrophy rate in AD and MCI than in normal individuals (Fox and Freeborough, 1997). Functional MRI also reflects synaptic dysfunction as FDG-PET (Sperling et al., 2011a). Our study reveals that functional MRI change exists only in NC group but not in MCI group with decreased FS of the visual occipital network, visual lateral network, and DMN but increased FS of the left frontoparietal network in high-risk subjects. In addition to memory impairment, visuospatial perception dysfunction is another prominent problem in AD and relevant cerebral functional changes occur in early stages of AD (Mandal et al., 2012). Previous studies revealed that visual occipital and visual lateral networks were related to higher order visual stimuli and complex stimuli, respectively, whereas the visual medial network was related to simple visual stimuli and higher order or complex visual processing is more vulnerable to aging (Habak and Faubert, 2000; Heine et al., 2012). Our results displayed a similar pattern in high-risk normal subjects, and therefore, visual processing networks are impaired more in relatively high-level systems and this phenomenon exists not only in the aging process but also in normal individuals with AD risk. In addition, symptoms related to visuospatial dysfunction occur in the AD stage but rfMRI is able to detect a relevant change in underlying RSN much earlier to normal status with high AD risk. DMN was noted to be impaired in patients with AD and MCI as compared with normal subjects (Greicius et al., 2004; Bai et al., 2008), and it was involved in many high-level cognitive functions, such as episodic memory, the well-known cognitive change in early AD, and the social cognitive process, which is also impaired in AD (Mevel et al., 2011; Cosentino et al., 2014). Our study further shows that decreased FS also occurs in normal people with high AD risk as compared with those with low risk, suggesting that the AD effect on DMN starts earlier from preclinical status. The significant differences of rfMRI between high- and low-risk subjects in our study occur only in NC group but not in MCI group, compatible with the findings of previous studies, in which rfMRI was with similar ability as FDG-PET to reflect abnormality earlier than structural MRI (Sperling et al., 2011a). Although both FDG-PET and rfMRI are a measure of synaptic activity, FDG-PET is focused on overall synaptic activity whereas rfMRI, especially in the network approach, primarily reflects integrated synaptic activity in some specific networks (Johnson et al., 2012). In our NC and MCI groups, FDG-PET shows persistently overall declined neuronal activity in both high-risk NC and MCI subjects, whereas rfMRI reveals only a neuronal activity change in high-risk NC subjects. A review of rfMRI in MCI and AD pointed out that the results of this modality in MCI are currently heterogeneous and even the condition of MCI status varies a lot (Dickerson and Sperling, 2008). Our results show that the rfMRI change might be more obvious in asymptomatic status than in MCI with already the presence of decreased activity and compensatory hyperactivity change in NC group. Moreover, impaired synaptic activity based on the network approach of rfMRI might have a different pattern of functional change in MCI status and is of lower power to identify high-risk MCI subjects as compared with the overall synaptic change in FDG-PET.

Within two-year follow-up, subjects with both higher T-tau/Aβ_42_ and P-tau/Aβ_42_ in NC or MCI were more likely to convert to MCI or AD, respectively. In addition, these two ratios were significantly higher in converters than in stable subjects in both NC and MCI groups. There results supported that these two ratios were important and reliable predictors for the possibility of conversion in the near future in NC and MCI statuses, further consolidating the above findings of risk evaluation based on these two ratios in our study. In addition, as compared with stable subjects in NC group, converters had characteristics of older age, lower FDG-PET value, lower Hippo/ICV ratio, higher brain atrophy, and increased FS of the sensorimotor network. Previous studies revealed that increased age and female were risk factors for AD (Inzelberg et al., 2015). Our results did not display gender effect but the risk of old age for AD was also noted in our NC group even though the included subjects were all elderly. Thus, age effect in the AD pathological process might be persistent and extend to even the age of 70–80, like our studying population. The lower FDG-PET value in converters was compatible with the results of high-risk subjects in our study, suggesting that this measurement was sensitive not only to selecting high-risk subjects but also to predicting future conversion. Although structural MRI did not display any difference between high- and low-risk subjects in NC, the converters had a lower Hippo/ICV ratio and higher brain atrophy. Considering that all the subjects in this study were relatively old, it was still reasonable to notice a structural change in converters from NC to MCI because the structural change started in late preclinical status (Jack et al., 2010). RfMRI revealed significantly higher FS of the sensorimotor network, which might be an early compensatory hyperactivity change, but this network was different from those identified in high-risk subjects. However, rfMRI was still able to reveal a significant change in real conversion from NC status. Initially, the sensorimotor network, a functional network with a lower cognitive level, was thought to be stable in MCI and AD, but lots of studies reveal that the alteration of its relevant functions, including the olfaction, hearing, visual, and motor systems, might precede the onset of cognitive impairments with being persistently worse when AD progresses. Therefore, the change of the associated functions becomes a strong risk factor for AD and functional impairment of the sensorimotor network was found in a later study (Wang et al., 2015). Our results further support that the sensorimotor network changes early in the AD process with already the presence of compensatory hyperactivity in converters from NC to MCI. As compared with stable subjects in MCI group, converters had characteristics of a lower FDG-PET value and decreased FS of the executive control network. Decreased FDG-PET was a persistently significant finding in our study, compatible with a previous study showing that this change extended along the continuum from normal cognitive status to MCI to AD (Minoshima et al., 1997). Our results further demonstrated that this change was able to reflect AD risk and to predict future conversion in NC and MCI status. Although no obvious rfMRI change was noted in high-risk MCI subjects, decreased FS of the executive control network in MCI converters was present, suggesting that rfMRI change could also extend along the continuum from normal cognitive status to MCI to AD. The executive control network is related to information processing in working memory, problem solving, and decision making, all of which are implicated in AD. This network was found to be slightly increased FC in MCI but sharply decreased in AD (Wang et al., 2015). The more obvious change of decreased FC in AD can be reflected in our results, and we further point out that the functional impairment might predominantly happen during the conversion from MCI to AD. The overall changes of rfMRI in our study do not reveal any single network with a persistently significant change in high-risk subjects of NC and MCI and in converters of NC and MCI. Functional MRI was also considered as the measurement of synaptic function like FDG-PET, and in our study, the rfMRI change was present in converters of both NC and MCI, compatible with FDG-PET results. However, this change was only found in high-risk NC subjects but not in high-risk MCI subjects, probably because the indicators of AD risk in our study were not perfect in MCI status. The ratios of tau and Aβ_42_ are highly but not completely correlated with amyloid deposition, or the amyloid deposition is already in a relatively stable status with less distinctness between truly high- and low-risk subjects in MCI (Jack et al., 2010; Fagan et al., 2011). Furthermore, using rfMRI networks to evaluate the AD pathological process should include multiple networks, whereas the overall FDG uptake can be more easy to reflect the AD pathological process without further acquiring detailed regional information even though previous studies suggested that specific regional decreased FDG uptake was present in AD (Jagust et al., 2007). The application of CSF biomarkers and their predictors for amyloid deposition in the AD pathological process can be reflected by FDG-PET, rfMRI, and structural MRI, all of which are less invasive than the necessary procedure of lumbar puncture to gain CSF samples. RfMRI is able to detect a network change in high-risk subjects in NC and converters in 2 years in NC and MCI. Structural MRI can demonstrate a change in high-risk MCI subjects and in converters from NC to MCI and from MCI to AD. The combination of functional and structural MRI is competitive to FDG-PET in the overall results.

In this study, some limitations should be announced. We use the average ratio of tau protein and Aβ_42_ to divide subjects into high- and low-risk subgroups in NC and MCI groups; however, this stratification is not quite good because the average ratio will vary a lot in another subject group and we still cannot define a specific ratio to separate high- and low-risk subjects with strong evidence in the literature. Thus, this method is only for the ease of statistical analysis. Except for rfMRI results, most values of parameters are from the ADNI database; it may lack some important information, such as regional uptake of FDG-PET. To solve these problems, we may use another database to verify our findings or wait for more subjects included from the ADNI database and a more reliable ratio of tau protein and Aβ_42_ to define high-risk subjects in future studies. The MMSE is not statistically different between NC and MCI groups, probably suggesting that the selection criteria of MCI subjects are not optimal.

## Conclusion

A combination of functional and structural MRI is safer and less expensive but with similar power as FDG-PET to reflect CSF change in the AD pathological process and to identify high-risk subjects and converters in NC and MCI. This combination might be a good screening tool for AD, but the complexity is of concern and how early this modality can identify subjects at risk for future conversion needs further evaluation. Our results reveal that it can predict future conversion within at least 2 years.

## Author Contributions

C-CH prepared this manuscript. W-MH and C-HC collected the data. Z-YJ analyzed the data. C-PL designed this study.

## Conflict of Interest Statement

The authors declare that the research was conducted in the absence of any commercial or financial relationships that could be construed as a potential conflict of interest. The reviewer CM and handling Editor declared their shared affiliation.

## Footnotes

1 http://fsl.fmrib.ox.ac.uk/fsl/fslwiki/
